# Has the COVID-19 pandemic influenced suicide rates differentially according to socioeconomic indices and ethnicity? More evidence is needed globally

**DOI:** 10.1017/S2045796022000543

**Published:** 2022-10-11

**Authors:** Roger T. Webb, Ann John, Duleeka Knipe, Lana Bojanić, Dana Dekel, Emily Eyles, Amanda Marchant, Faraz Mughal, Jane Pirkis, Lena Schmidt, David Gunnell

**Affiliations:** 1 Division of Psychology and Mental Health, The University of Manchester, Manchester, UK; 2 National Institute for Health and Care Research Greater Manchester Patient Safety Translational Research Centre (NIHR GM PSTRC), Manchester, UK; 3 Swansea University Medical School, Swansea University, Swansea, UK; 4 Public Health Wales, Cardiff, UK; 5 Population Health Sciences, Bristol Medical School, University of Bristol, Bristol, UK; 6 National Institute for Health and Care Research Applied Research Collaboration West (NIHR ARC West), University Hospitals Bristol and Weston NHS Foundation Trust, Bristol, UK; 7 School of Medicine, Keele University, Keele, UK; 8 Centre for Mental Health, Melbourne School of Population and Global Health, The University of Melbourne, Melbourne, Australia; 9 Sciome LLC, Research Triangle Park, Durham, NC, USA; 10 National Institute for Health and Care Research Biomedical Research Centre, University Hospitals Bristol and Weston NHS Foundation Trust and the University of Bristol, Bristol, UK

**Keywords:** Economic issues, epidemiology, social factors, suicide

## Abstract

The COVID-19 pandemic has harmed many people's mental health globally. Whilst the evidence generated thus far from high-income countries regarding the pandemic's impact on suicide rates is generally reassuring, we know little about its influence on this outcome in lower- and middle-income countries or among marginalised and disadvantaged people. There are some signals for concern regarding the pandemic's potentially unequal impact on suicide rates, with some of the affected demographic subgroups and regions being at elevated risk before the pandemic began. However, the evidence-base for this topic is currently sparse, and studies conducted to date have generally not taken account of pre-pandemic temporal trends. The collection of accurate, complete and comparable data on suicide rate trends in ethnic minority and low-income groups should be prioritised. The vulnerability of low-income groups will likely be exacerbated further by the current energy supply and cost-of-living crises in many countries. It is therefore crucial that reassuring messaging highlighting the stability of suicide rates during the pandemic does not lead to complacency among policymakers.

## The unequal adverse impact of the COVID-19 pandemic

By September 2022 over 600 million people had been infected by SARS-CoV-2 and approximately 6.5 million had died from COVID-19 (Worldometer). Excess mortality from all causes during the pandemic has been greatest amongst ethnic minority groups and deprived communities (Barnard *et al*., 2021; Stokes *et al*., 2021). The infectious disease itself, along with societal restrictions implemented to control the virus's spread and subsequent economic fallout, have impacted negatively on many people's mental health globally (Santomauro *et al*., 2021). The mental health of individuals in ethnic minority and low-income groups has been particularly badly affected in some countries (Maffly-Kipp *et al*., 2021; Pierce *et al*., 2021).

Despite the pandemic's evident harmful mental health impact, a recent analysis of suicide rates in 33 nations (and regions of countries) over the first 9–15 months of the pandemic revealed little evidence of heightened suicide risk (Pirkis *et al*., 2022), although case counts were greater than expected in some places and for certain age/sex groups. However, temporal trends in suicide rates among ethnic minority groups could not be examined due to a lack of granularity in the reported results. Such an assessment is crucial because social marginalisation (Di Thiene *et al*., 2015) and material deprivation (Rehkopf and Buka, 2006; Li *et al*., 2011) are known to be major determinants of suicide risk at the population level.

Utilising published outputs identified by our ‘living systematic review’ of suicide and suicidal behaviour during the pandemic (John *et al*., 2020), this editorial summarises the relatively small volume of evidence indicating an unequal impact of the pandemic on risk of dying by suicide among ethnic minority groups and in people on low incomes and between areas according to their varying levels of social deprivation.

## Emerging evidence of divergent temporal trends in suicide rates according to socioeconomic indices and ethnicity since the start of the pandemic

Indicative evidence for the unequal impact of the pandemic on suicide rates across socioeconomic and ethnic groups has thus far been generated from a small set of countries. Much of the evidence has emanated from the USA, including three state-wide investigations, in Connecticut (Mitchell and Li, 2021), Maryland (Bray *et al*., 2021) and Michigan (Larson and Bergmans, 2022), and a national analysis (Ehlman *et al*., 2022). Each of these studies was conducted using individual-level data and each revealed reductions in suicide rates among White Americans in 2020, with no evidence of similar falls in ethnic minority groups during the pandemic's first year. Between 2019 and 2020 the suicide rate among all US residents fell by 3%, with the largest reduction (4.5%) found among non-Hispanic White people (Ehlman *et al*., 2022). However, in this national study, rates in Hispanic males and non-Hispanic multiracial females increased by 29 and 6%, respectively. Rates also increased by 10% among American Indian and Alaska Native males – groups with the highest suicide rates in the US prior to the pandemic, although the small size of this ethnic group meant that statistical evidence for the rise was insubstantial. Other methodological limitations of these US studies included broad ethnicity categorisation (Mitchell and Li, 2021), inconsistent patterns by ethnicity found across the whole observation period (Bray *et al*., 2021), and crude comparison of annual rates between two single adjacent years without consideration of prior temporal trends (Ehlman *et al*., 2022).

Japan is one of relatively few high-income countries where the national suicide rate rose during the pandemic. It increased by 16% during the second infection wave in late 2020 (Tanaka and Okamoto, 2021). With two temporal trend graphs plotted separately providing a crude ecological correlational indication, Horita and Moriguchi (2022) reported that suicide and unemployment rates both rose concurrently in Japan during the pandemic's second wave. Given what is known about the hugely damaging impact of previous economic downturns on suicide rates (Barr *et al*., 2012; Reeves *et al*., 2012), the looming threat of recession in many countries globally, and its potential for widening existing inequities in suicide risk, is particularly concerning.

Evidence for temporal trends in suicide rates in lower- and middle-income countries during the pandemic is limited, which is a major concern given that roughly four in five of all suicides worldwide occur in these nations (Knipe *et al*., 2022). Some evidence of varying temporal trends in suicide risk according to ethnicity and socioeconomic indices has emerged (Pirkis *et al*., 2022), including two studies conducted in lower-middle income countries (India: Arya *et al*., 2022; Nepal: Archarya *et al*., 2022) and another two studies carried out in upper-middle income countries (Brazil: Orellana and de Souza, 2022; Ecuador: Gerstner *et al*., 2022). For three of these studies ecological analyses were conducted using information on socioeconomic indices aggregated regionally (Archarya *et al*., 2022; Arya *et al*., 2022; Orellana and de Souza, 2022), and therefore their reported findings must be interpreted cautiously (Piantadosi *et al*., 1988).

In India, annual suicide rates were already rising prior to the pandemic, but the increase observed during 2020 (18% in males; 5% in females) was greater compared to preceding rate rises. This increase was particularly evident among males in low sociodemographic index (SDI) states and among males and females in the high SDI states (Arya *et al*., 2022). In Nepal, the largest increases in suicide rates across the first 15 months of the pandemic were observed in two of the nation's poorest provinces with low human development indices as well as a large volume of seasonal migrant workers (Archarya *et al*., 2022). Between March and December 2020, the national suicide rate in Brazil fell by 13%, but substantial excess suicide risks were observed in some age and sex groups in the nation's more deprived provincial regions. Thus, a 26% excess of suicides occurred among men aged 60 years and older in the Northern region, and in the North-Eastern region there was a 40% excess in women aged 60 years and older (Orellana and de Souza, 2022). In Ecuador, there was no rise in frequency of all police-reported suicides between March 2020 and June 2021, with a proportional decrease in suicides observed among indigenous people and other ethnic minority groups (Gerstner *et al*., 2022).

## Recommendations for developing a complete evidence-base for the pandemic's unequal impact on suicide risk

Developing such an evidence-base that is both robust and comprehensive is an urgent priority that will in due course inform post-pandemic suicide prevention strategies. It is crucial that the currently incomplete and inconsistent patchwork of evidence regarding the pandemic's demographically uneven impact on suicide risk is augmented and strengthened. This will entail utilising what is now historic routinely collected interlinked registry data in some countries, such as the Scandinavian nations. In other countries, novel data linkages will be required to expedite these investigations. In many of the poorest parts of the world, due to an absence of good quality population-based data, the true extent to which the pandemic heightened pre-existing mental health inequalities, including suicide risk, is unknown at this time. Investigators ought to prioritise conducting high-quality local studies in the absence of population-based linked datasets in these countries.

During 2020 and 2021, many researchers tended to examine temporal trends in suicide case counts or rates using brief time periods in line with dynamic fluctuations in SARS-CoV-2 infection rates or imposition *v*. lifting of societal restrictions. Consequently, some investigations were statistically underpowered for identifying short-term variability in trends according to ethnic or socioeconomic subgroups. As researchers retrospectively develop comprehensive evidence of the pandemic's potentially uneven demographic impact on suicide rates, considerably longer time periods should be examined to maximise statistical power. Other longstanding impediments that are not specific to the pandemic epoch include inaccurate or incomplete recording of ethnicity in electronic health records (Gomez *et al*., 2005) and in cause-specific mortality records, delays in assignment of suicide as a cause of death due to protracted coronial procedures (Cui *et al*., 2004), and a scarcity of population-based datasets that would enable multilevel modelling of suicide risk at individual, household and neighbourhood levels.

The role of socioeconomic differences in driving divergent regional temporal trends in suicide rates during the pandemic according to their levels of urbanicity–rurality also requires elucidation. The complex relationship between population density and poverty is likely to vary greatly between different parts of the world. Thus, the greatest falls in suicide rates in the USA during 2020 occurred in large metropolitan urban centres, whereas rates did not fall in the nation's predominantly rural regions (Ehlman *et al*., 2022). By contrast, in that year higher population density predicted suicide rate increases across Mexico's 32 states, with approximately twice as many suicides occurring in Mexico City than the expected value (Borges *et al*., 2022). Similarly, in Ecuador, the proportion of suicides occurring in urban and coastal regions rose (Gerstner *et al*., 2022).

## Conclusion

Although the evidence-base for this topic is limited, there are some clear early signals that the pandemic's impact on suicide risk has been variable according to ethnicity and socioeconomic indices. This indicates that policymakers will need to implement financial safety nets and other mitigatory measures (Gunnell *et al*., 2020; Shand *et al*., 2022) to protect the most vulnerable individuals, families and communities during what seems likely to be troubled and turbulent post-pandemic era. This is especially important given the current challenges to global mental health posed by climate change and extreme weather events, economic instability and stagnation, civil unrest, armed conflict between nations and threats to the supply of affordable food and energy. There may be no universally consistent patterns of uneven impact on suicide risk because the extent and severity of COVID-19, the measures implemented to prevent the virus's spread and the economic protection measures have differed greatly between different parts of the world as well as between and within countries.

Accurate and complete coding of ethnicity and socioeconomic indices in mortality records and in other linkable routinely collected datasets should be prioritised. As much as is possible, coding and classification of such information ought to be standardised internationally. Real-time surveillance (RTS) of suicide rates is also urgently needed in all countries (Baran *et al*., 2021), perhaps starting at municipality level initially in countries where resources are relatively scarce and digital infrastructures are not advanced. Newly developed RTS systems will require sufficient granularity for identifying high-risk demographic subgroups whilst informing rapid evidence-based policy responses, which can be a challenging trade-off. Public agencies also need to monitor temporal trends in ‘deaths of despair’ from drug overdoses and alcohol misuse as well as suicide (Arena *et al*., 2020).
